# Peripheral artery disease in diabetes

**DOI:** 10.3389/fendo.2026.1811582

**Published:** 2026-04-20

**Authors:** Sheena Amin, Eri Fukaya, Anand Athavale

**Affiliations:** Division of Vascular Surgery, Department of Surgery, Stanford University School of Medicine, Palo Alto, CA, United States

**Keywords:** diabetes, diabetes mellitus, PAD, peripheral artery disease, type 2 diabetes

## Abstract

Peripheral artery disease in diabetes mellitus represents a distinct clinical entity characterized by diffuse distal arterial disease, medial calcification, microvascular dysfunction, neuropathy, and a pro-thromboinflammatory milieu. These features contribute to atypical presentations, diagnostic challenges, accelerated progression, and elevated risks of limb loss and cardiovascular events. Management requires integrated strategies encompassing metabolic optimization, lifestyle modification, pharmacotherapy, antithrombotic therapy, supervised exercise, and timely revascularization. Emerging approaches, including GLP-1 receptor agonists, SGLT2 inhibitors, and regenerative therapies, show promise in improving vascular, functional, and limb outcomes. Advancing outcomes in diabetic peripheral artery disease will depend on mechanism-driven screening, individualized therapies, and novel interventions to prevent complications.

## 1 Introduction

Peripheral artery disease (PAD), a severe form of atherosclerotic cardiovascular disease, carries a substantial and increasing global burden, contributing significantly to disability, limb amputation, and mortality (1). Type 2 diabetes mellitus (T2D) markedly elevates the risk of PAD, with affected individuals exhibiting a more than twofold higher prevalence compared to the general population (2). A recent systematic review reported 12.5%–22% prevalence of PAD in individuals with T2D (3). The presence of these comorbid conditions results in more aggressive disease progression, greater severity, and poorer clinical outcomes than in non-diabetic patients. This translates to poorer quality of life, increased healthcare resource use and costs (3). These epidemiologic insights highlight the need for heightened vigilance, early detection, and tailored management strategies in this high-risk population. In this review, we summarize and critically evaluate the current state of knowledge on PAD in T2D, highlighting key advances, ongoing debates, and emerging opportunities for improved outcomes.

## 2 Pathophysiology

Beyond atherosclerosis, emerging genetic and mechanistic studies highlight roles for calcific, inflammatory, and angiogenic defects in PAD development. In T2D, these features are amplified by chronic hyperglycemia, metabolic derangements, endothelial dysfunction, persistent inflammation, and neuropathy, collectively accelerating atherosclerosis, promoting more aggressive disease, and producing a distinct, often more severe clinical phenotype than in non-diabetic individuals (4).

### 2.1 Pro-inflammatory state and accelerated atherosclerosis

Chronic hyperglycemia in T2D promotes a proinflammatory, proatherogenic milieu through interconnected mechanisms that amplify oxidative stress, endothelial dysfunction, and vascular inflammation. Excess glucose drives reactive oxygen species (ROS) generation via mitochondrial dysfunction, glucose auto-oxidation, polyol pathway flux, and advanced glycation end-product (AGE) formation, leading to vascular cell injury and lipid peroxidation (5). AGE–RAGE signaling and hyperglycemia-induced activation of protein kinase C (PKC) further enhance cytokine release, endothelial permeability, and expression of adhesion molecules such as VCAM-1 and ICAM-1, facilitating leukocyte recruitment to the vessel wall (6).

Recruited monocytes differentiate into macrophages that avidly internalize modified lipoproteins, including oxidized or glycated low-density lipoprotein (LDL) and triglyceride-rich lipoprotein remnants, via scavenger receptors upregulated by hyperglycemia and impaired insulin signaling, accelerating foam cell formation (7). Hyperglycemia also disrupts the balance between vasodilatory and vasoconstrictive mediators and directly enhances platelet activation and aggregation, reinforcing a prothrombotic, inflammatory environment that accelerates plaque progression and instability, contributing to the aggressive atherosclerotic phenotype observed in diabetes (6).

### 2.2 Thrombosis and hypercoagulability

Diabetes creates a vascular and hemostatic environment that strongly predisposes patients with PAD to thrombotic complications and adverse limb outcomes (8). Atherosclerotic plaques in diabetic arteries exhibit greater instability, with larger lipid-rich necrotic cores, increased inflammatory infiltration, and reduced fibrous cap integrity, increasing susceptibility to plaque disruption and exposure of thrombogenic substrates such as collagen and tissue factor (9).

Platelet function is intrinsically altered in diabetes, with abnormalities in surface receptor expression and intracellular signaling that lower the activation threshold. Enhanced signaling through pathways involving glycoprotein IIb/IIIa, P2Y_1__2_, and thromboxane A_2_ promotes exaggerated adhesion, degranulation, and aggregation (10). Accelerated platelet turnover results in a greater proportion of reticulated, hyperreactive platelets that may be less responsive to antiplatelet therapy (11). Structural membrane changes driven by lipid remodeling and protein glycation further increase platelet responsiveness and endothelial adhesion (12).

Endothelial dysfunction is a central contributor to diabetic hypercoagulability. Hyperglycemia-induced oxidative stress reduces nitric oxide bioavailability and degrades the endothelial glycocalyx, impairing critical antithrombotic and anti-inflammatory properties of the vascular surface (13). Endothelial cells exhibit increased tissue factor expression and reduced activity of endogenous anticoagulant pathways, shifting the balance toward coagulation (14). These changes promote rapid initiation of thrombin generation following plaque disruption or endothelial injury.

Diabetes is also associated with quantitative and qualitative abnormalities of coagulation and fibrinolytic systems. Elevated levels of fibrinogen and procoagulant factors, combined with reduced tissue factor pathway inhibitor, amplify thrombin generation and fibrin formation (15). Thrombin-mediated factor XIII activation enhances fibrin cross-linking, producing mechanically stable clots. Increased plasminogen activator inhibitor-1 suppresses fibrinolysis, while glycation and oxidation of fibrinogen and plasminogen alter fibrin architecture. Incorporation of antifibrinolytic proteins creates denser fibrin networks that resist endogenous clot breakdown (16).

Additional vascular dysfunction arises from hemoglobin glycation, which disrupts nitric oxide transport and release, impairing vasodilation and further promoting platelet activation and inflammation. Collectively, these abnormalities create a sustained prothrombotic state characterized by enhanced clot formation and reduced clot resolution, contributing to higher rates of arterial occlusion, restenosis, and adverse limb events in patients with diabetes and PAD (4).

In addition to vascular structural changes, diabetes fosters a pro-thrombotic state. NF-κB activation, cytokine release (IL-6, TNF-α), leukocyte-endothelial adhesion, and platelet hyperreactivity collectively create a thrombo-inflammatory milieu that accelerates plaque formation, destabilization, and occlusion (17, 18). Hypercoagulability is clinically evident in diabetic PAD, as demonstrated by increased thrombin generation, higher clot strength on thromboelastography, and elevated fibrinogen levels, particularly in patients with poor glycemic control.

The presence of Factor V Leiden (F5 p.R506Q) has also been associated with PAD and advanced phenotypes such as chronic limb-threatening ischemia (CLTI) and amputation, underscoring a genetic contribution to thrombotic risk (19).

### 2.3 Medial arterial calcification and distal disease

**Figure 1:**
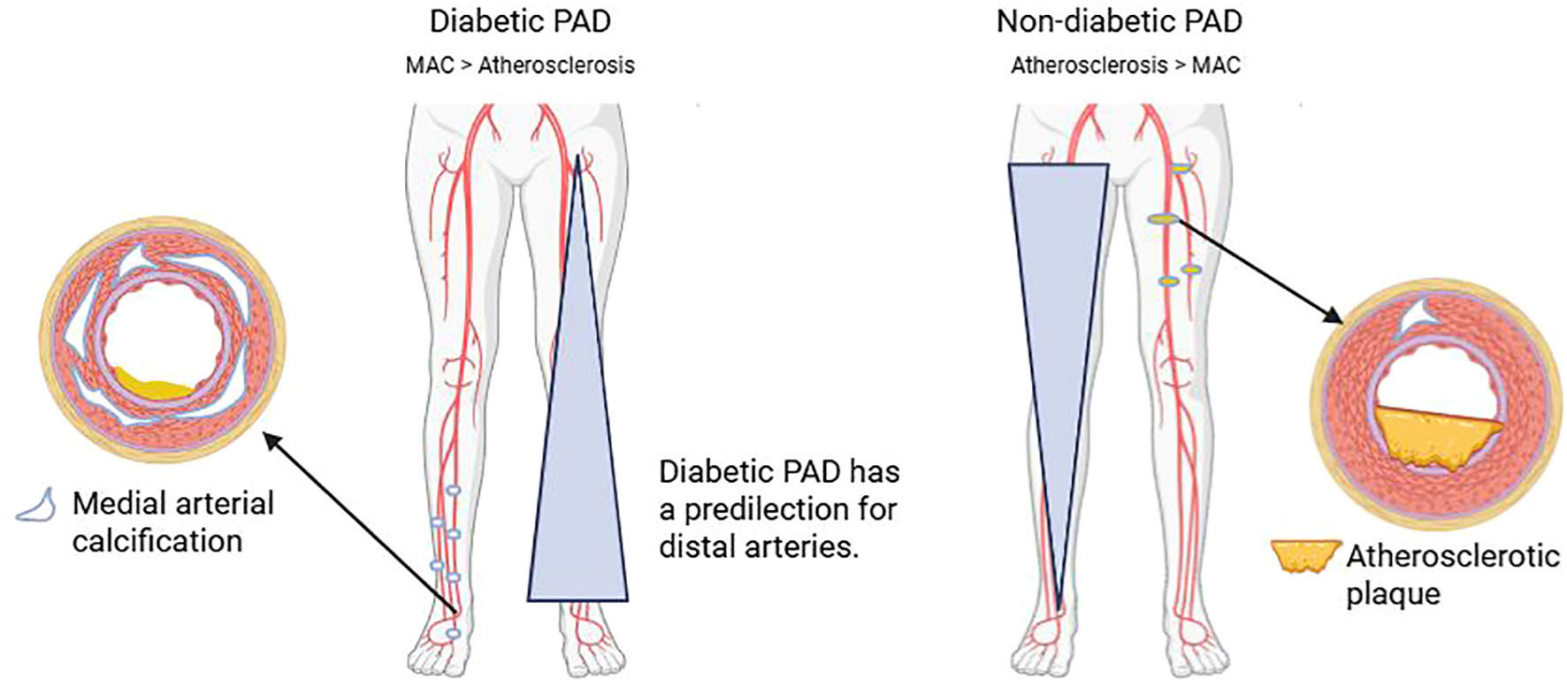
Distribution of PAD burden in diabetics versus non-diabetics.

Medial arterial calcification (MAC) is highly prevalent in patients with diabetes, particularly in the distal tibial and pedal arteries, where it contributes to increased arterial stiffness, impaired distal perfusion, and important diagnostic limitations, including falsely elevated ankle–brachial index (ABI) values and non-compressible vessels (20). Pathologic analyses further highlight key differences between distal and proximal PAD; in a study by Narula et al., infrapopliteal arteries in patients with critical limb ischemia demonstrated a predominance of medial calcification with relatively less lipid-rich atherosclerotic plaque compared with proximal vessels, supporting the concept that distal disease is driven more by arterial stiffening and calcification than by classic atheroma formation (21) (**Figure 1**). Histologically, MAC is characterized by calcium phosphate deposition within the medial layer of the arterial wall and represents an actively regulated process rather than passive mineral accumulation (22).

At the cellular level, vascular smooth muscle cells (VSMCs) undergo osteogenic phenotypic transformation, marked by upregulation of bone-associated transcription factors such as RUNX2 and osterix, increased expression of alkaline phosphatase, and production of bone matrix proteins, all of which facilitate hydroxyapatite deposition within the extracellular matrix (23). This process is promoted by inflammatory signaling pathways and oxidative stress, which enhance VSMC apoptosis and generate membrane-bound vesicles that serve as nucleation sites for calcium crystal formation (23). Concurrent downregulation of endogenous inhibitors of calcification, including matrix Gla protein and fetuin-A, further permits unchecked mineral deposition within the medial layer (24, 25).

Disordered mineral metabolism, frequently present in patients with diabetes and often exacerbated by concomitant chronic kidney disease, further accelerates medial calcification. Elevated extracellular phosphate stimulates sodium-dependent phosphate transporters in VSMCs, activating osteogenic gene programs and promoting intracellular mineral accumulation (26). These molecular changes drive progressive medial stiffening and loss of arterial compliance.

Functionally, MAC increases pulse wave velocity and reduces vasodilatory reserve, impairing distal perfusion and limiting the capacity of peripheral arteries to augment blood flow in response to metabolic demand. In PAD, these effects worsen tissue ischemia, impair wound healing, and contribute to CLTI. Interventional challenges include heavily calcified infrapopliteal vessels resisting balloon expansion, limiting drug delivery, and increasing dissection and elastic recoil, leading to lower technical success and higher restenosis rate (27).

Clinically, diabetes is associated with a markedly increased burden of medial calcification compared with non-diabetic populations, and the frequent coexistence of proximal atherosclerotic disease, distal medial calcification, and microvascular dysfunction creates a uniquely severe and diffuse vascular phenotype. This combination of impaired inflow, poor distal runoff, and compromised microcirculatory perfusion substantially limits the durability and clinical benefit of revascularization and contributes to persistently high risks of limb loss in diabetic PAD.

### 2.4 Microvascular dysfunction

Diabetic PAD is characterized by microvascular impairment that further compromises tissue perfusion, even in the presence of moderate macrovascular obstruction. Chronic hyperglycemia leads to capillary rarefaction, impaired autoregulation, endothelial barrier dysfunction, and diminished collateral vessel formation (28). This results in early tissue ischemia, delayed wound healing, and susceptibility to ulceration and infection, contributing to the high rates of major adverse limb events (MALE) observed in diabetic PAD. Neuropathy further masks typical PAD symptoms, delaying recognition until advanced stages with foot ulcers, gangrene, or acute limb ischemia (2). Together, microvascular dysfunction, impaired perfusion, diminished tissue repair, neuropathy-related loss of protective sensation, and structural foot changes such as muscle atrophy and Charcot arthropathy create an environment prone to repetitive trauma, skin breakdown, and progressive ulceration, contributing to high rates of MALE in diabetic PAD (29).

### 2.5 Genetic and metabolic contributors

Recent advances in genetic studies have begun to clarify the heritable component underlying susceptibility to PAD, particularly in individuals with T2D. A 2023 systematic review and meta-analysis identified more than 230 DNA variants associated with PAD across 112 studies; however, only a small subset, notably ICAM1 (rs5498), IL6 (rs1800795), and LIPC (rs2070895), demonstrated consistent, reproducible associations across cohorts (30). In addition, a large genome-wide association study using data from the Million Veteran Program identified multiple loci associated with PAD, including variants in genes such as *LPA*, *F5*, and *CHRNA3*, implicating pathways related to lipid metabolism, thrombosis, and smoking behavior. These findings provided important insights into shared genetic mechanisms linking PAD with cardiometabolic risk factors while also highlighting PAD-specific biological pathways (31).

In addition to common variants, trans-ethnic gene-based analyses have revealed extensive genetic overlap between T2D and PAD, identifying several shared genes, including ANKFY1, STARD10, AP3S2, KCNJ11, and KCNQ1, that appear to mediate vascular dysfunction across diverse ancestries (32). These discoveries support the view that diabetes and PAD do not coexist merely because they share clinical risk factors, but because they share underlying genetic pathways governing endothelial dysfunction, inflammation, and metabolic stress.

Metabolic dysfunction further amplifies this genetic susceptibility. Insulin resistance and chronic hyperglycemia in T2D drive endothelial dysfunction through reduced nitric oxide bioavailability and increased oxidative stress and inflammation. Together with AGE-RAGE signaling, dyslipidemia, and disordered mineral metabolism, these processes accelerate atherosclerosis, medial arterial calcification, and impaired angiogenesis (23, 33).

The interplay of polygenic risk, insulin resistance, hyperglycemia, lipid abnormalities, mineral metabolism derangements, and endothelial/microvascular dysfunction creates a “double hit” in diabetes. This explains why diabetic PAD is earlier, more distal, more diffuse, and linked to higher rates of ulceration, infection, MALE, and amputation. Recognizing this dual genetic-metabolic landscape supports strategies such as earlier screening in genetically high-risk patients, precision-guided therapy, and development of agents targeting calcification, oxidative stress, and impaired angiogenesis.

## 3 Clinical features and diagnostic challenges

### 3.1 Atypical presentation

Diabetic PAD is often underrecognized due to neuropathy, which blunts classic claudication or rest pain symptoms. Patients may remain asymptomatic until presenting with foot ulcers, gangrene, or acute limb ischemia. Distal and diffuse disease further complicates the clinical picture, as proximal pulses may remain palpable while distal perfusion is severely impaired.

### 3.2 Diagnostic modalities

Traditional ABI measurements are frequently unreliable in diabetic PAD due to MAC, which renders arteries non-compressible and leads to falsely elevated readings (34). Alternative assessments such as the toe-brachial index (TBI) provide improved accuracy for detecting distal disease, as digital arteries are less affected by calcification (35). Functional assessments, including transcutaneous oxygen pressure (TcPO_2_) and skin perfusion pressure (SPP), can be valuable for predicting wound healing and amputation risk, although they are difficult to clinically implement (36). Imaging modalities, including duplex ultrasound, computed tomography angiography (CTA), and magnetic resonance angiography (MRA), offer detailed anatomical visualization, allowing identification of multilevel and distal disease to guide revascularization strategies. Emerging AI-based imaging analyses and circulating biomarkers of endothelial dysfunction and inflammation may enhance early detection and risk stratification (37).

## 4 Natural history and prognosis

Diabetic PAD is associated with accelerated progression and worse clinical outcomes compared with non-diabetic PAD (2). Rates of CLTI, non-healing ulcers, infection, minor and major amputations, and cardiovascular events (myocardial infarction, stroke) are all significantly higher in this population (3). Mortality is also elevated, reflecting the systemic burden of atherosclerosis and thrombo-inflammation (36). Medial arterial calcification has been shown to double amputation risk in diabetic foot ulcer patients and more than quadruple major amputation risk in those with concomitant chronic kidney disease (38, 39). These observations highlight the need for aggressive, multifactorial management in diabetic PAD.

## 5 Management strategies

Guideline-directed PAD therapy includes risk factor modification with statins, blood pressure control, and smoking cessation, along with antiplatelet therapy to reduce major adverse cardiovascular events (MACE) and low-dose antithrombotic therapy to reduce both MACE and MALE (40). Cilostazol is used for claudication symptom relief, and supervised exercise therapy improves walking distance and functional status. ACC/AHA Class I recommendations include smoking cessation, exercise therapy, statins, aspirin, clopidogrel, and cilostazol, though cilostazol has not been shown to reduce cardiovascular or all-cause mortality; Class IIa recommendations include ezetimibe and PCSK9 inhibitors (41).

### 5.1 Supervised exercise therapy

Supervised exercise therapy (SET) is a cornerstone of non-invasive management for PAD patients with intermittent claudication. Structured, physician-supervised programs improve walking distance, functional capacity, and quality of life by promoting collateral vessel development and enhancing endothelial function. In the CLEVER study, which randomized patients to SET, stent revascularization, or optimal medical care, both SET and stenting significantly improved peak treadmill walking time at 18 months compared with medical therapy alone (42).

### 5.2 Antithrombotic therapy

Evidence from the VOYAGER trial and thrombotic studies supports the use of low-dose anticoagulation in addition to antiplatelet therapy for high-risk diabetic PAD, particularly post-revascularization, to reduce both cardiovascular and limb ischemic events (43). Hypercoagulability in diabetes, driven by thrombin generation, platelet hyperreactivity, and fibrin formation, provides a mechanistic justification for this approach.

### 5.3 Revascularization

Revascularization, via endovascular or surgical approaches, is indicated for patients with significant symptomatic PAD or CLTI. However, distal and heavily calcified lesions, particularly in diabetic patients, pose substantial technical challenges, leading to higher rates of restenosis, procedural failure, and repeat interventions. Factors such as poor distal targets, limited vessel compliance, and complex lesion morphology further reduce the durability of conventional revascularization. Careful pre-procedural planning, including advanced imaging, functional assessments, and patient selection, is essential to optimize procedural outcomes.

For patients who are not candidates for conventional endovascular or surgical interventions, emerging therapies offer alternative strategies for limb salvage. One such approach is transcatheter arterialization of deep veins, in which arterial blood is redirected into the venous system to perfuse ischemic tissue. Recent studies, including the PROMISE II trial, have demonstrated that this technique can improve wound healing, reduce the risk of major limb amputation, and provide a meaningful treatment option for “no-option” patients with advanced distal disease (44). While further studies are needed to refine patient selection and optimize procedural protocols, transcatheter arterialization represents a promising treatment for patients with severe, diffuse, or otherwise untreatable PAD.

### 5.4 Emerging pharmacotherapies

#### 5.4.1 GLP-1 receptor agonists

The most important advance has come from the STRIDE trial, the first large-scale randomized, placebo-controlled trial evaluating a glucose-lowering agent specifically in symptomatic PAD. In 792 patients across 20 countries, semaglutide 1.0 mg once weekly for 52 weeks significantly improved maximal walking distance (MWD), with an estimated treatment ratio (ETR) of 1.13 (95% CI 1.06–1.21; p = 0.0004) (45). Absolute improvements reached ~26 m (median) and ~40 m (mean), surpassing clinically meaningful thresholds for claudication therapy.

Secondary endpoints favored semaglutide, including improved pain-free walking distance, VascuQoL-6 scores, and reduced claudication symptoms. Benefits were consistent across subgroups stratified by HbA1c, BMI, diabetes duration, or background therapy, suggesting vascular and microvascular effects beyond glycemic control. However, the authors note that translating improvements in treadmill-based walking distance into meaningful changes in everyday function remains challenging, highlighting an important limitation of the trial.

Mechanistically, GLP-1 receptor agonists are hypothesized to improve endothelial function, attenuate oxidative stress and inflammatory signaling, and potentially enhance microvascular perfusion and angiogenesis. If these vascular effects are confirmed, the therapeutic benefit of semaglutide may extend beyond glycemic lowering and could theoretically apply to patients with peripheral artery disease independent of diabetes type, including those with type 1 diabetes. However, these mechanisms remain incompletely established and require confirmation in dedicated vascular outcome studies (45).

Liraglutide (STARDUST trial): The STARDUST trial provided supportive biologic evidence for GLP-1 RA benefit. In this randomized study of 55 patients with T2D and PAD, liraglutide up to 1.8 mg daily improved TcPO_2_, reduced inflammatory markers, and increased 6-minute walking distance at 6 months. An 18-month follow-up demonstrated sustained perfusion improvements and favorable angiogenic marker profiles (46). Though underpowered, STARDUST strengthened the rationale for GLP-1–based therapies in PAD.

#### 5.4.2 SGLT2 inhibitors

SGLT2 inhibitors provide robust cardiovascular and renal benefits, but limb-specific effects are less clear (47). Randomized cardiovascular outcome trials have not consistently reported PAD outcomes, and patients with severe CLTI were often excluded. Although early CANVAS data suggested increased amputation risk with canagliflozin, the FDA removed the boxed warning in 2020 after evidence indicated overall benefit outweighed risk (48).

Clinicians still monitor for foot ulcers, infections, ischemia, or prior amputation when initiating therapy. Combined therapy with GLP-1 RAs and SGLT2 inhibitors, now common in T2D management, may provide complementary metabolic, hemodynamic, endothelial, and microvascular benefits for PAD, though dedicated trials are needed.

#### 5.4.3 Lipid-lowering therapies

LDL-cholesterol-lowering therapies, including statins and PCSK9 inhibitors, have demonstrated consistent reductions in macrovascular PAD events and MALE. For example, PCSK9 inhibition with evolocumab significantly reduced MALE in the FOURIER trial, and a prespecified analysis of the ODYSSEY OUTCOMES trial showed reduced PAD events with alirocumab on a background of intensive statin therapy (49, 50).

Fibrates, which activate PPARα, have been evaluated with exploratory evidence suggestive of benefit in PAD-related endpoints. In the FIELD trial, fenofibrate was associated with reduced risk of non-traumatic lower-extremity amputations, particularly minor amputations without known large-vessel disease (51). Additionally, an exploratory analysis of the PROMINENT trial found that pemafibrate was associated with a 37% relative reduction in incident lower-extremity ischemic ulceration and gangrene in patients with type 2 diabetes on background statin therapy (52).

Lipoprotein(a) [Lp(a)] has emerged as an important lipid-related risk factor in PAD, with elevated levels independently associated with increased incidence and severity of PAD as well as major adverse limb outcomes (53).

Therapeutic agents targeting Lp(a), including antisense oligonucleotides and small interfering RNAs, have shown substantial Lp(a) lowering in clinical trials, and large outcomes studies are underway to determine whether Lp(a) reduction translates to reduced cardiovascular and limb events (54).

Taken together, these data underscore the multifaceted role of dyslipidemia in PAD pathogenesis, spanning from LDL-C and Lp(a)-mediated macrovascular risk to potential microvascular and pleiotropic influences mediated by PPARα pathways, and highlight the evolving landscape of lipid-modifying therapies that may further mitigate limb-related complications in diabetic PAD.

#### 5.4.4 Regenerative and mechanism-targeted therapies

Beyond glucose-lowering agents, regenerative medicine approaches, including autologous stem cell therapy and angiogenic cell injections, are being explored to address diffuse disease that is often not feasible for revascularization in diabetic PAD (55). Early-phase trials demonstrate potential improvements in ulcer healing, skin perfusion, and limb salvage, though evidence remains heterogeneous and underpowered (55).

Therapies targeting underlying mechanisms, such as vascular calcification inhibitors, AGE–RAGE antagonists, antioxidants, or endothelial-repair agents, are under development and may ultimately complement antithrombotic or metabolic therapy in high-risk diabetic PAD populations (56, 57).

#### 5.4.5 Anti-inflammatory therapies

Beyond its role in PAD pathogenesis, inflammation represents a promising therapeutic target. The CANTOS trial demonstrated that IL-1β inhibition with canakinumab reduced systemic inflammation and suggested potential benefits on peripheral vascular outcomes in patients with PAD, though sample sizes were small and the trial was primarily designed for cardiovascular events (58).

Similarly, IL-6 signaling has been strongly linked to cardiovascular and peripheral vascular events, as highlighted in analyses from the CIRT trial (59). Targeted IL-6 inhibition is being actively investigated in PAD populations; for example, the ZEUS trial is evaluating the monoclonal antibody ziltivekimab to determine whether reducing IL-6–mediated inflammation can improve vascular outcomes and reduce complications in patients with PAD (60).

Collectively, these studies support the potential for anti-inflammatory strategies to complement current therapies in PAD, though safety, cost, and definitive clinical benefit remain important considerations.

## 6 Discussion

Despite recent advances, significant gaps remain in the care of diabetic PAD. Optimal strategies for screening asymptomatic disease, particularly approaches that integrate functional and anatomical assessments, still require validation. Emerging biomarkers and AI-based imaging tools offer the potential for earlier detection and more precise risk stratification, while the comparative effectiveness of endovascular versus surgical interventions for diffuse, distal disease, the role of cardiometabolic therapies such as GLP-1 receptor agonists and SGLT2 inhibitors, and strategies to enhance wound healing and limb salvage remain areas of active investigation. Addressing these gaps is critical to reducing both limb-related and cardiovascular morbidity in this high-risk population.

PAD in diabetes represents a distinct clinical entity, characterized by diffuse distal disease, medial arterial calcification, microvascular dysfunction, neuropathy, and a pro-thromboinflammatory environment. These features contribute to atypical clinical presentations, diagnostic challenges, accelerated progression, and elevated risks of limb loss and cardiovascular events. Accurate diagnosis often requires a combination of functional and anatomical testing, while effective management necessitates an integrated approach including metabolic optimization, lifestyle modification, pharmacotherapy, antithrombotic strategies, and timely revascularization. Emerging therapies, such as GLP-1 receptor agonists, SGLT2 inhibitors, lipid-lowering agents, anti-inflammatory interventions, and regenerative approaches, hold promise for improving both vascular and limb outcomes.

The excess burden of PAD in type 2 diabetes stems from the convergence of metabolic dysfunction, vascular inflammation, and renal disease. Chronic hyperglycemia, dyslipidemia, and hypertension drive endothelial injury, atherogenesis, and plaque progression, while diabetic nephropathy and chronic kidney disease further promote vascular calcification, arterial stiffness, and impaired distal perfusion. These processes culminate in diffuse, multilevel disease with poorer revascularization outcomes and faster progression to critical limb-threatening ischemia. Importantly, diabetic patients develop PAD at younger ages and progress more rapidly than non-diabetic individuals, underscoring the need for early detection, vigilant risk factor management, and timely interventions. With advances in precision therapeutics, innovative pharmacologic agents, and emerging regenerative strategies, there is now real potential to transform the management of diabetic PAD, reducing limb loss, improving functional outcomes, and ultimately altering the disease trajectory.
